# Identification of latent profiles of social isolation based on structural and functional aspects

**DOI:** 10.1093/geroni/igaf122.3111

**Published:** 2025-12-31

**Authors:** Dokyung Yoon, Elizabeth Zelinski, Teal Eich

**Affiliations:** University of Southern California Leonard Davis School of Gerontology, Los Angeles, California, United States; University of Southern California, Los Angeles, California, United States; University of Southern California, Los Angeles, California, United States

## Abstract

Social isolation negatively impacts health, and incorporating multidimensional indicators into its measurement may better capture its complexity. This study examined latent profiles of older adults based on social isolation indicators and explored how various factors are associated with profile membership. Participants included 5,466 older adults aged 65 + (Mage = 75.16) from the 2012 and 2014 waves of the Health and Retirement Study. Social isolation was measured through both structural (contact frequency with children/other family members/friends and social participation) and functional (loneliness and social support) aspects. Furthermore, the study incorporated sociodemographic, psychological, environmental, and health-related factors as covariates. Latent Profile Analysis and logistic regression were conducted using Mplus. Results exhibited five social isolation group profiles: (1) ‘highly socially engaged’ (6%), (2) ‘moderately socially engaged’ (20%), (3) ‘moderately isolated, but not lonely’ (46%), (4) ‘socially active but unsupported’ (12%), and (5) ‘most socially isolated’ (16%). Structural and functional aspects of social isolation were generally aligned. However, the presence of the ‘socially active but unsupported’ group suggests that engagement in social activities does not necessarily equate to strong social support. Lower education levels were consistently associated with less social engagement across groups and men tended to belong to socially isolated groups more than women. Compared to the ‘highly socially engaged’ group, all other groups showed lower levels of extraversion and openness. The ‘moderately isolated, but not lonely’ and ‘most socially isolated’ groups exhibited worse cognition, more chronic conditions, and greater depressive symptoms. The findings reveal distinct typologies of social isolation among older adults.

